# A multi-device and multi-operator dataset from mobile network coverage on Android devices

**DOI:** 10.1016/j.dib.2024.111146

**Published:** 2024-11-16

**Authors:** Vandermi Silva, Gabriel Tavares, Carlos Freitas, Felipe Maklouf, Chavdar Ivanov, Raimundo Barreto, Rosiane de Freitas

**Affiliations:** a Institute of Computing, Federal University of Amazonas, Av. Gen. Rodrigo Octávio, 6200 Setor Norte do Campus Universitário - Coroado, Manaus, AM, Brazil; b ICET Institute of Exact Science and Technology, Federal University of Amazonas, R. Nossa Sra. do Rosário, 3863 - Tiradentes, Itacoatiara, AM Brazil; c Motorola Mobility, SP-340, Km 128 - 7 - Tanquinho Velho, Jaguariúna, SP, Brazil

**Keywords:** Mobile network coverage, Network performance, Radio parameters, Signal strength, 4G LTE technology

## Abstract

The demand for mobile coverage with adequate signal quality has triggered criticism due to the maturity of the Internet's diffusion in today's society. However, with the deployment of 5G networks, even 5G NSA by 4G LTE, the complexity of the operating environment of mobile networks has increased. To evaluate the behavior of mobile networks in terms of signal quality and other important metrics for mobile telephony, we developed a dataset consisting of 33 radio parameters that can collect up to 736,974 records generated daily by smartphones and tablets. The dataset comprises samples collected in cities situated on the banks of the Amazon and Negro rivers. To create the dataset, an application was designed for the Android operating system using the Kotlin programming language, which can collect data in real time and generate a CSV file. After post-processing the collected data with data science techniques, the filtered dataset was stored in the Mendeley public repository. We divided the data into three regions: the metropolitan area of Manaus, the middle Solimões River, and the middle Amazonas River. To improve the performance of the experiments, the database was separated according to the cities and locations collected.

Specifications Table

**Table:** 

Subject	Computer Science, Data Science, Engineering, Computer Engineering, Telecommunications.
Specific subject area	Embedded Systems, Network Performance, Telecommunications.
Type of data	Raw, Analyzed, Filtered
Data collection	The dataset has 10 datasets collected from 9 cities located on the Amazon and Negro Rivers. The complete database covering all regions has 33 columns and 736,974 rows. In addition to the primary dataset, we divided the data into three regions: the metropolitan area of Manaus, the middle Solimões River, and the middle Amazonas River. During the scheduled trips, data were collected along rivers and roads that provide access to the locations selected for the experiment. The data was processed, indexed, and organized into a comprehensive dataset, then categorized by location. This organization allows experiments using the entire dataset across all cities or with data specific to an individual city.
Data source location	Institution: Federal University of Amazonas, Manaus-Amazonas Country: Brazil Latitude and longitude: 3°05′17.3"S 59°57′52.7"W
Data accessibility	Repository name: Mendeley Data Data identification number: DOI: 10.17632/tjg9426ykr.2 Direct URL to data: https://data.mendeley.com/datasets/tjg9426ykr/2 To access the database and conduct initial experiments, Python scripts were developed alongside the database to facilitate data loading and the generation of histograms and charts necessary for the initial investigation. In addition to the graph generation scripts, we also created heat maps based on the collected network variables. These scripts can be run using the Jupyter Notebook, found at [1]. The scripts are in the “src” folder, and the datasets are in the “network_dataset” folder. In addition, a folder containing the latest version of a network data collection application, named Armadeira, was made available in the “armadeira_app” folder and published in the Mendeley repository. Alternatively, users can use Google Colaboratory, an online programming tool, to access these resources remotely without needing local installation. The files must remain within the same directory for proper functionality.
Related Research Article	None

## 1 Value of the Data

• The data presented provides information regarding the main variables of mobile telephone networks collected in the Amazon region using a mobile application called Armadeira, which was developed by the research team. While there are other datasets with comparable attributes, it is notable that the Amazon region exhibits a low population density, particularly in the northern areas, where settlements are widely dispersed, lacking access to paved roads and frequently relying on river transportation. Consequently, the collection and storage of data from these specific regions is vital for the future provision of reliable telecommunications services in regions with such characteristics.
• The collected data was used to evaluate network parameters related to signal quality variables, latitude and longitude, signal transmission power, and signal-to-noise ratio, among other important parameters for measuring network quality. These parameters may assist other researchers in extracting insights into mobile network quality conditions, thereby enabling measurements to be made using the smartphone itself with the installed collection application, which was developed as part of this research project.
• The data helps analyze the mobile network parameters in any country because the collection application is internationalized and follows 3GPP standards.
• The data helps evaluate mobile network signal coverage based on 3G, 4G LTE, and 5G NSA network technology. A significant number of cities in Brazil and around the world have yet to adopt 5G technology. In such cases, a comprehensive assessment of existing network technologies is crucial for identifying areas with limited network coverage and characterizing the technologies in use at data collection sites.
• By evaluating quality parameters, heat maps can be created to identify network behavior based on location data, date and time of collection, and several quality metrics. This information is stored in a public database and it is freely available. In addition, heat maps based on quality parameters can help identify areas with poor mobile network coverage.
• The data is also valuable for applying Machine Learning techniques such as regression, decision tree, clustering, and others to develop predictive models for better assessing mobile network signal quality.

## 2 Background

Mobile network performance is a key factor in ensuring connectivity in remote regions, such as the Amazon rainforest. Despite the growing importance of telecommunications infrastructure [2], detailed data on network coverage in these areas is still lacking. Other impacting factors are the large distances between urban centers, the density of the forest, the non-linearly varying climate, and the various communication technologies (3G, 4G LTE, and 5G) used to cover urban and rural areas [3], which make data capture and normalization a challenge. It is also known that some previous studies have focused predominantly on urban environments [[4], [5], [6]], leaving a significant knowledge gap regarding signal quality in more challenging geographic locations.

This dataset was collected as part of the SWPERFI [7], research and development project, with the aim of evaluating mobile network performance in the state of Amazonas, especially in the regions close to the Solimões and Negro rivers. The data provides in-depth insights into several signal quality indicators, including RSSI, RSRP, RSRQ, RSNR, and CQI, under different conditions and transport modes. By addressing these gaps, the dataset offers valuable insights for academic researchers and telecommunications providers, which can guide the optimization of network infrastructure in the Amazonas and similar environments. The methodologies employed ensure the robustness and diversity of the data, making it a crucial resource for advancing the study and application of mobile network technologies.

This work presents several significant points that illustrate the importance of the dataset, both in terms of technological advancement and social and scientific impact. The development of the Armadeira application as a customized tool for real-time data collection represents an innovative technical solution, allowing for continuous and detailed analysis of signal quality in different environments. Furthermore, the regional impact, with the division of data between metropolitan areas and more remote regions, allows for a detailed comparative analysis that highlights the discrepancy between urban and rural regions in terms of network coverage. For instance, the data demonstrates that in urban areas, such as Manaus, coverage is relatively consistent, whereas in riverside areas, signal quality exhibits considerable fluctuations, impeding communication for isolated communities. This information can inform public policies aimed at expanding telecommunications infrastructure in under-served regions.

By making the dataset available in a public repository, other researchers will be able to replicate the experiences in similar contexts, such as rainforest regions in Africa or Southeast Asia, where geographical conditions impose similar challenges to network coverage. This will facilitate international collaboration and the development of a global body of knowledge on mobile networks in remote environments.

The dataset provides a wealth of information that can be leveraged to enhance the performance of mobile networks in challenging environments. Researchers can utilize this data to train machine learning algorithms that automatically adjust cell towers to optimize signal strength in areas with low coverage. A specific study could employ this information to develop dynamic spectrum allocation models, thereby maximizing coverage efficiency in isolated regions. This illustrates the potential of the dataset to facilitate the improvement of mobile network services.

Thus, the main contribution of this dataset is the integration of detailed data on signal quality in a challenging environment, which allows us to evaluate and optimize the performance of mobile networks in remote regions. This is key to overcoming previous limitations in predominantly urban studies and promoting connectivity in less accessible areas.

## 3 Data Description

The data utilized to construct the dataset presented in this study were collected as part of the research and development project, Software Intelligent Performance (SWPERFI). The experiments were conducted between August 2023 and April 2024 with the objective of investigating the relationship between specific mobile network variables that influence the quality of signal coverage in the Amazonas state region, encompassing areas in close proximity to the Solimões and Negro rivers. The data set is quantitative in nature, comprising the collection of network signal parameters, including the Received Signal Strength Indicator (RSSI), Reference Signal Received Power (RSRP), Reference Signal Received Quality (RSRQ), Reference Signal to Noise Ratio (RSNR), and Channel Quality Indicator (CQI), among others. The data were stored in text files in Comma-Separated Values (CSV) format and hosted on a cloud platform. To ensure the diversity and robustness of the data set, six smartphones from different manufacturers and models were utilized. To ensure the anonymity of the brands, the devices were identified numerically, from one to six, in the “model” field during the data pre-processing stage. The data was collected via a variety of means, including on foot, by river, and by land, using the Armadeira app installed on each of the project's smartphones. All of the smartphones were equipped with fully charged batteries and chips from the two main operators in the region, operating on 4G LTE and 5G NSA technologies. Table 1 presents the data dictionary for the set, which describes all of the variables that were collected during the experiments with their respective examples, Table 2 shows the extract of part of the dataset with location data sampling.

**Table 1:** Variables name, units, and feature description of the dataset “df_anonymized.csv” published in the Mendeley dataset. This table presents all the information about the variables collected from the exposed APIs of the Android OS, which are completely independent of the operator and manufacturer.

Feature Name	Type	Measure	Description
Model	string	NA	Indicates the device model for example, different manufacturers and types of smartphones (this feature has been anonymized). Example of received data “Moto G 200”: Note, this data was anonymized in the dataset.
Current Date Time	string	NA	Indicates the date and time the data was generated or logged. Example of received data “12/1/2023 7:54:35 AM”.
Iso	string	NA	Acronym of the country in which the data was collected. Example of received data Example of received data “BR”.
Location Status	boolean	NA	(TRUE) indicates if the user's location is enabled, (FALSE) indicates disabled. Example of received data “TRUE”.
Latitude	double	NA	The latitude value of the user's location. Example of received data “-2.69622”.
Longitude	double	NA	The longitude value of the user's location. Example of received data “-59.6932”.
Mobile Status	boolean	NA	(TRUE) indicates if the mobile device is turned on, (FALSE) indicates it is turned off. Example of received data “TRUE” .
Network Operator	int	NA	Identifies the mobile network operator to which the mobile device is camped (this feature has been anonymized). Example of received data “1”.
Network Type	string	NA	A parameter that indicates the type of mobile network currently in use by the mobile device, eg: 5G NSA, 4G, 3G, and 2G. Example of received data “4G”
dBm	float	decibels milliwatts	Received signal power in decibels relative to one milliwatt (dBm). Example of received data “-109”
RSRP LTE	float	decibels milliwatts	Reference signal received power (RSRP) in dBm. The average power received from a single Reference signal and its typical range is around -44dbm (good) to -140dbm (bad). Example of received data “-108”
RSSI LTE	float	decibels milliwatts	Received Signal Strength Indication in decibels relative to one milliwatt (RSSI).Example of received data “-81”
RSRQ LTE	float	decibels	Reference signal received quality (RSRQ) in dB. Indicates the quality of the received signal, and its range is typically -19.5dB (bad) to -3dB (good).Example of received data “-10”
SNR LTE	float	decibels	Reference to the signal-to-noise ratio of the given signal in dB. Example of received data “6”
CQI LTE	int	NA	Channel quality indicator - a measure of the quality of the communication channel. Example of received data “10”.
ASU Level LTE	int	NA	Arbitrary Strength Unit (ASU) is an integer value proportional to the received signal strength measured by the mobile phone. Example of received data “24”.
PCI LTE	int	NA	Physical cell identity (PCI) of the current mobile network cell to which the device is connected. Example of received data “184”.
CI LTE	int	NA	Cell identity (CI) of the current mobile network cell to which the device is connected. Example of received data “95198211”.
TAC LTE	int		Tracking Area Code (TAC) is used to identify a Carrier's Tracking Area. Example of received data “55397”.
RX	long	bytes	Received data rate in bytes. Example of received data “96”.
TX	long	bytes	Transmitted data rate in bytes. Example of received data “1536”.
Downstream Bandwidth	int	Kbps	The data download speed on a mobile network is measured in kilobits per second (Kbps). Example of received data “82650”.
Upstream Bandwidth	int	Kbps	The data upload speed on a mobile network is measured in kilobits per second (Kbps). Example of received data “1419879”.
Level	int	NA	Abstract level value for overall signal strength. A single integer from 0 to 4 represents the overall signal quality. This can be considered as a result of many different radio technology inputs. 0 represents very weak signal strength, while 4 represents very strong signal strength. Example of received data “1”.
EARFCN	int	NA	E-UTRA Absolute Radio Frequency Channel Number (EARFCN) - a unique identifier for a radio channel in a cellular network. Example of received data “3350”.
Band	int	NA	The frequency band on which the mobile device is operating. Example of received data “7”.
Frequency	int	MHz	The frequency in megahertz (MHz) at which the mobile device operates on a cellular network. Example of received data “2600”.
Downlink Frequency	float	MHz	downlink frequency channel. Example of received data “2680”.
Uplink Frequency	float	MHz	uplink frequency channel. Example of received data “2560”.
MCC	string	NA	Mobile country code (MCC) - a unique identifier assigned to a country for mobile network purposes. Example of received data “724”.
MNC	string	NA	Mobile network code (MNC) - a unique identifier assigned to a mobile network within a country. Example of received data “2560”.
Chip Operator	int	NA	Identifies the operator of the SIM card in the mobile device. Example of received data “2”. Note, this data was anonymized in the dataset.
Roaming	boolean	NA	This allows a network user to obtain connectivity in areas outside the geographic location where they are registered. In the database, TRUE indicates that the user is roaming and FALSE suggests that he is not. Example of received data “FALSE”.

**Table 2:** The dataset shows the extract of part of the dataset with location data sampling, collected from applications running on Android devices, is a part of the dataset, filtered to select latitude and longitude.

Model	Current Date Time	Latitude	Longitude
1	2023-08-21 12:55:21	-3.04213	-60.0062
1	2023-12-07 20:08:42	-3.06602	-60.0131
1	2023-11-22 02:58:07	-3.04264	-60.0071
1	2024-03-26 12:57:49	-3.04265	-60.0071
3	2024-02-06 12:17:29	-3.08836	-59.9649
3	2024-04-08 20:56:56	-3.04263	-60.0007
3	2024-04-08 14:01:33	-3.04266	-60.0007

## 4 Experimental Design, Materials and Methods

Several studies present datasets collected from mobile networks in 4G and 5G technologies, as referenced in the literature [[8], [9], [10], [11], [12], [13]]. These datasets include KPI indicators for different operators, synthetic data, and contextual information, all aimed at enhancing the performance of 4G and 5G networks. The collected data is used to create datasets that, in turn, are used for measuring mobile network features on a large scale, providing critical information to ensure a better user experience by understanding the mobile network behavior in urban environments characterized by high population densities and various mobility patterns.

On the other hand, studies are dedicated to developing emulated datasets to support the automation of 5G networks, as presented in [11]. In these cases, multiple resources, variations of signal strength, and collected radio metrics were used to create deployment and testbed scenarios to support creating emulated network traffic and comparing it with real traffic from commercial operators' networks. To complement the dataset, the authors provided a synthetic dataset generated from network simulators to provide additional evidence about competition between mobile phones camped on the same cell to provide the user with final information that would otherwise not be available without eNodeB.

Despite the greater use in telecommunications, 5G networks are also being used to develop indoor and outdoor wireless control solutions. However, performance problems persist as real-time algorithms still cannot improve it effectively, despite the widespread use of Machine Learning algorithms to solve various issues. This does not occur in New Radio (5G NR) applications because more specific datasets are needed [12]. Thus, the authors in [12] proposed two methods based on Optimization Modulo Theories (OMT) and Satisfiability Modulo Theories (SMT) to generate training datasets for 5G NR scheduling.

In [13], the concepts of 5G networks are presented in terms of the criticality in development, analysis, and security, as well as examples regarding the deterioration of network properties, a summary of the protocol, and the semantic analysis of the specifications themselves, which, according to the authors, it is done manually. The authors created a public dataset for Natural Language Process (NLP) research with security-related text classification to reduce manual effort. These texts protect properties that can be used in communication protocol testing.

Therefore, this work aims to provide real data on the variables and mobile network KPIs collected using our tool and make them available to the academic community to understand the quality characteristics of the mobile network signal in the Amazon region. The work describes the data and explains its use cases by the community, provides initial studies on how exploratory analysis can be applied, and offers a link to access the databases and the initial scripts used, making them available in an open-source format. We emphasize that this is a fundamental study on network variables correlating signal quality with data collection independent of operators and mobile devices.

The following topics present the structure of the repository, how it is stored on the mobile device and sent to the cloud, as well as the complete process of data collection, processing and storage.

### 4.1 Data repository

**Fig. 1:**
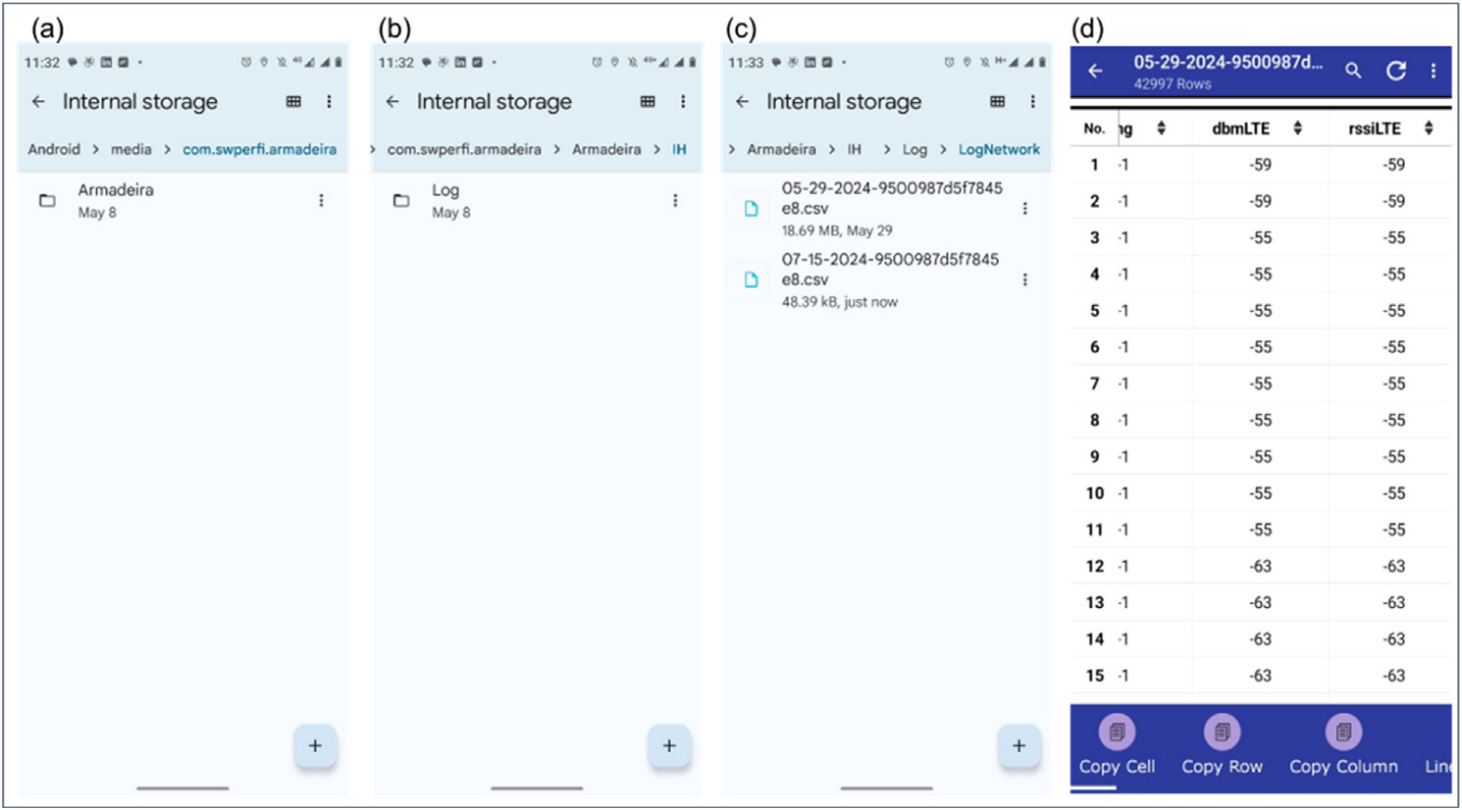
Internal storage data collection embedded in a mobile device. The data is temporarily stored in the Android's internal storage in the “Armadeira/IH/Log/LogNetwork” directory until the collection is complete, after which it is automatically sent to the cloud.

**Fig. 2:**
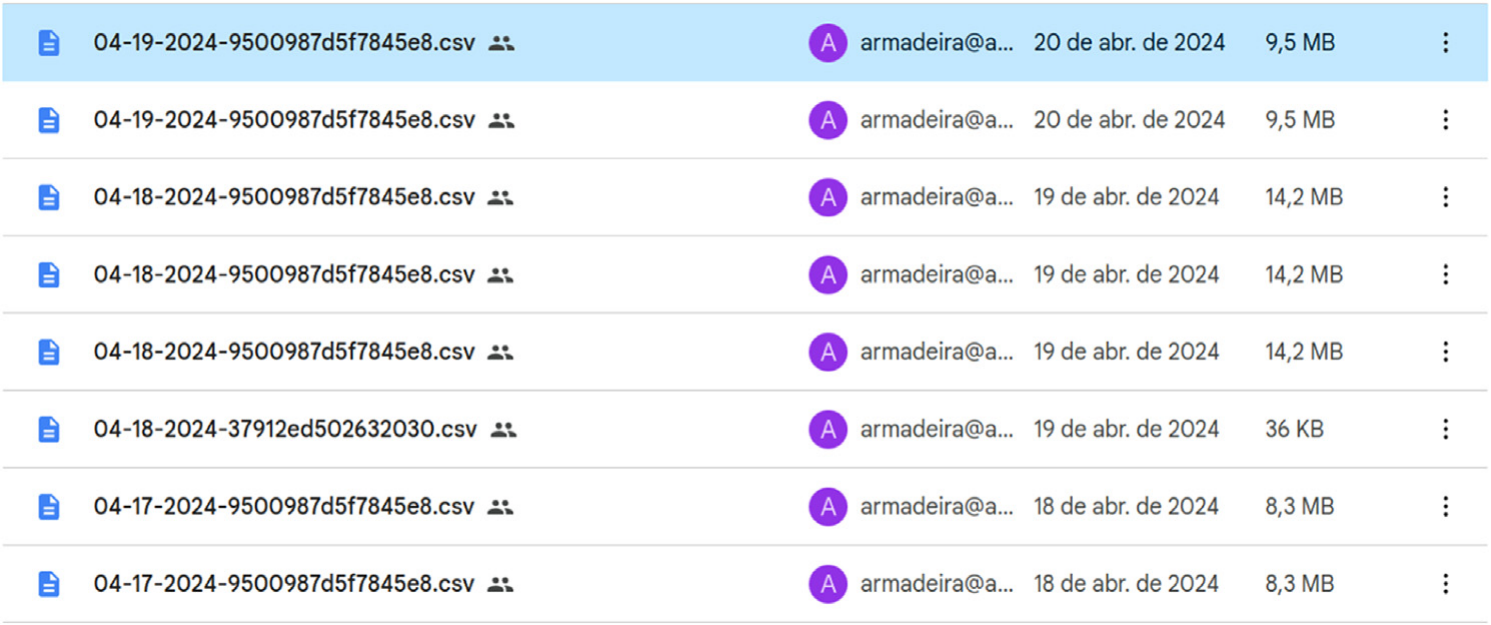
The Armadeira application automatically sends the dataset to the cloud. After sending the data, a CSV file is generated. The filename comprises the collection date and time concatenated with the unique device identifier.

The database for this article is available in the Mendeley Data Repository [14]. The data is organized in a folder named “network_dataset,” which contains a list of datasets. Each dataset is named according to the device ID concatenated with the timestamp at which it was collected. Fig. 1 shows the raw dataset stored inside the mobile device, and Fig. 2 shows the dataset stored in the cloud after the preprocessing steps. The collected data contains mobile network variables such as Reference Signal Received Power (RSRP), Reference Signal Received Quality (RSRQ), Signal-to-Noise Ratio (SNR), and Channel Quality Indicator (CQI), collected in real-time and stored on the mobile device in Comma-separated Values (CSV) data format. After completing the daily collection, the device automatically sends the file to the cloud. Fig. 1 shows the distribution of records in Android devices and illustrates the internal organization of files stored on the mobile device before being sent to the cloud. Fig. 1a depicts the “Armadeira” application folder, including the “log” subfolder depicted in Fig. 1b and the stored CSV files in Fig. 1c. Fig. 1d presents an extract of the file contents.

This dataset was constructed by continuously gathering data from various manufacturers' devices running the Android OS, which were connected to the networks of different mobile operators. Data collection was facilitated using the Armadeira Application during regular device usage.

The application was developed with the aim of quickly collecting data from the mobile network, taking inspiration from the Armadeira spider, a species endemic to the Amazon region and known for its speed and efficiency. Data is collected from the mobile network on a daily basis, with the application automatically performing collection functions in the background, with an interval of one second between collections. Specific threads run at each time slot to collect data as a function of location and time. This data is grouped into data objects and then converted into a CSV file format that is stored on the device during a daily collection. After this process, the CSV file is automatically transferred to the cloud and then deleted from the device.

The application is compatible with Android 12 or higher and requires 2GB of RAM and 150MB of free space.The application's architectural design adheres to the Model-View-ViewModel (MVVM) pattern, which is used in the main module called “App” to differentiate and assign responsibilities among the Model, View, and ViewModel components.

**Fig. 3:**
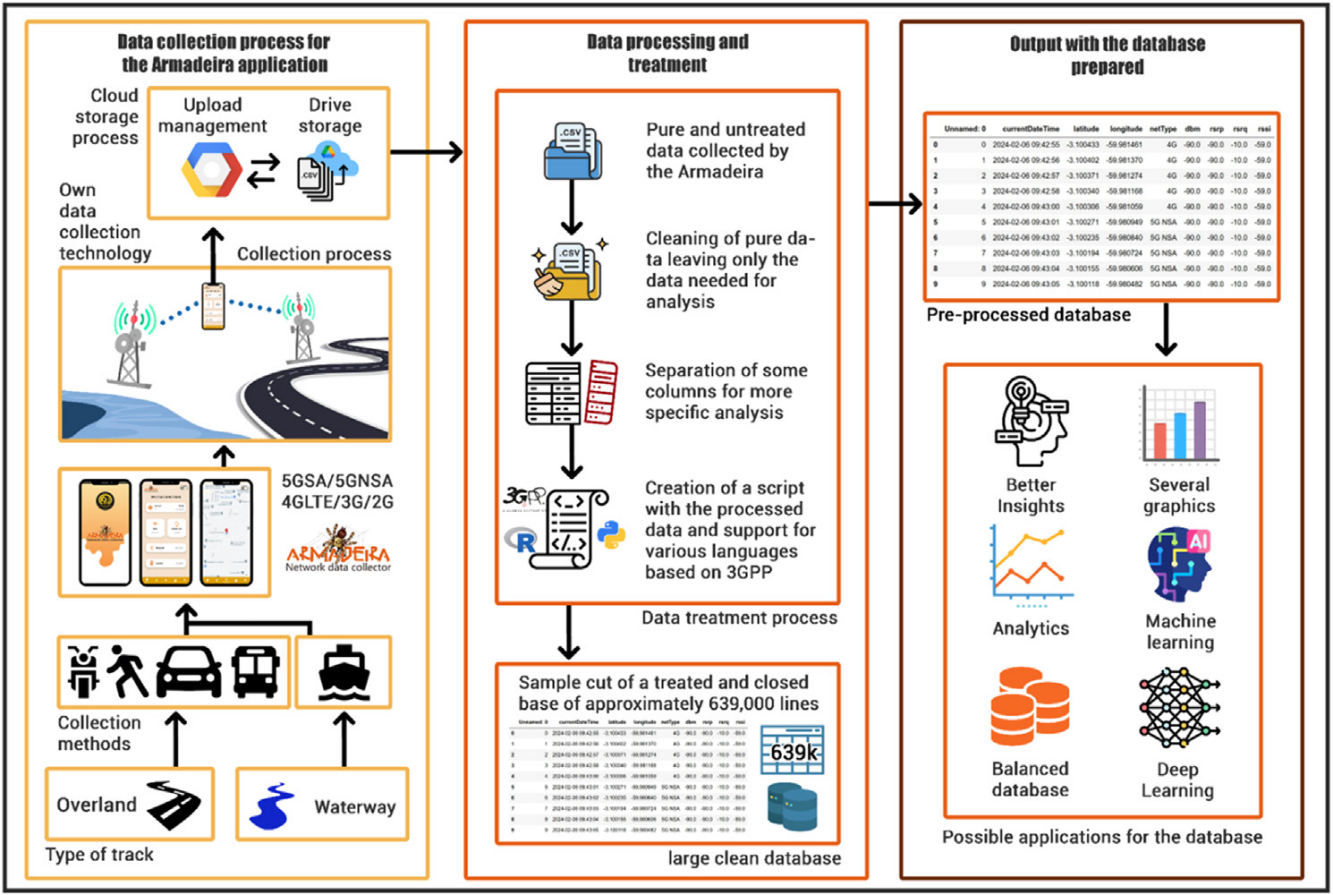
The flow of the data collection method covers the three main phases: the data collection process for the Armadeira application, data processing and treatment, and output with the pre-processed dataset.

The Domain module is designed to centralize all operations related to variable collection, computation performance, and resource management. This includes DTOs (Data Transfer Objects), Armadeira-specific models and utilities. This model facilitates a clear delineation of resources, thereby improving code maintainability and extensibility. Fig. 3 provides a high-level overview of the data collection methodology.

### 4.2 Data collection methodology

The methodology for collecting and utilizing the data was divided into three parts: (1) data collection process through the Armadeira application developed by the research team; (2) Data processing and data treatment, applying data science tools; and (3) Output, resulting in data prepared to be used in possible data analytics, machine learning, and deep learning applications. The details of the entire process will be presented below.

### 4.3 Data collection process for the Armadeira application

The transportation and communication limitations in the Amazon region are constant due to the large number of rivers and forests and the hot and humid climate, with large amounts of rain and storms. On the other hand, there are few roads connecting the cities. It was, therefore, necessary to adapt the data collection process from the mobile networks that are the subject of this paper to cover the areas of interest through waterway transportation and road transportation. The data was collected using smartphones with the Armadeira mobile app installed while traveling through the main cities chosen for the experiments. The application collects the variables of the 5G No Stand Alone 5G NSA, 4G Long-Term Evolution LTE, 3G, and 2G networks. The application stores them internally on the smartphone and, at the end of the collection, automatically sends the data in CSV format to be processed in the cloud. For the first part of the process, the technologies used were the Kotlin programming language for the Android Operating System and the Google Data Cloud for external storage. The application collects the variables of the 5G Non-Stand Alone (NSA), 4G Long-Term Evolution (LTE), 3G, and 2G networks.

### 4.4 Data processing and treatment

In the second part of the process, the data sent to the cloud is parsed and processed to identify outliers, missing data, and other inconsistencies that may have occurred during storage. At this stage, the data undergoes evaluation using scripts written in either Python or R programming languages, and the network variables are scrutinized for compliance with the 3rd Generation Partnership Project (3GPP) standard.

During this phase, significant columns were isolated for specific analyses and verified using Python scripts, resulting in a pre-processed dataset. The Python programming language and 3GPP documentation were utilized during this stage of the process. Next, Python libraries such as Pandas and Glob were used to merge all the CSV files stored in the cloud into a single, larger file, which combined data collected at different times and locations. Each entry included a timestamp, coordinates, and mobile network variables. The dataset was then cleaned to remove outliers and handle missing data. The Z-score method [15], a statistical technique that quantifies the position of a data point relative to the overall mean of a dataset, expressed in terms of standard deviations, was employed to detect and remove outliers. This method is useful for identifying values significantly above or below the mean, as well as for standardizing data to facilitate comparisons across different datasets. To address missing data, we applied the Backward Fill (BFILL) technique [16], which propagates the last non-zero value forward. This approach is particularly useful in time series where data continuity is crucial. We chose this method due to the small number and scattered nature of missing values in our data.

### 4.5 Output with the pre-processed dataset

**Fig. 4:**
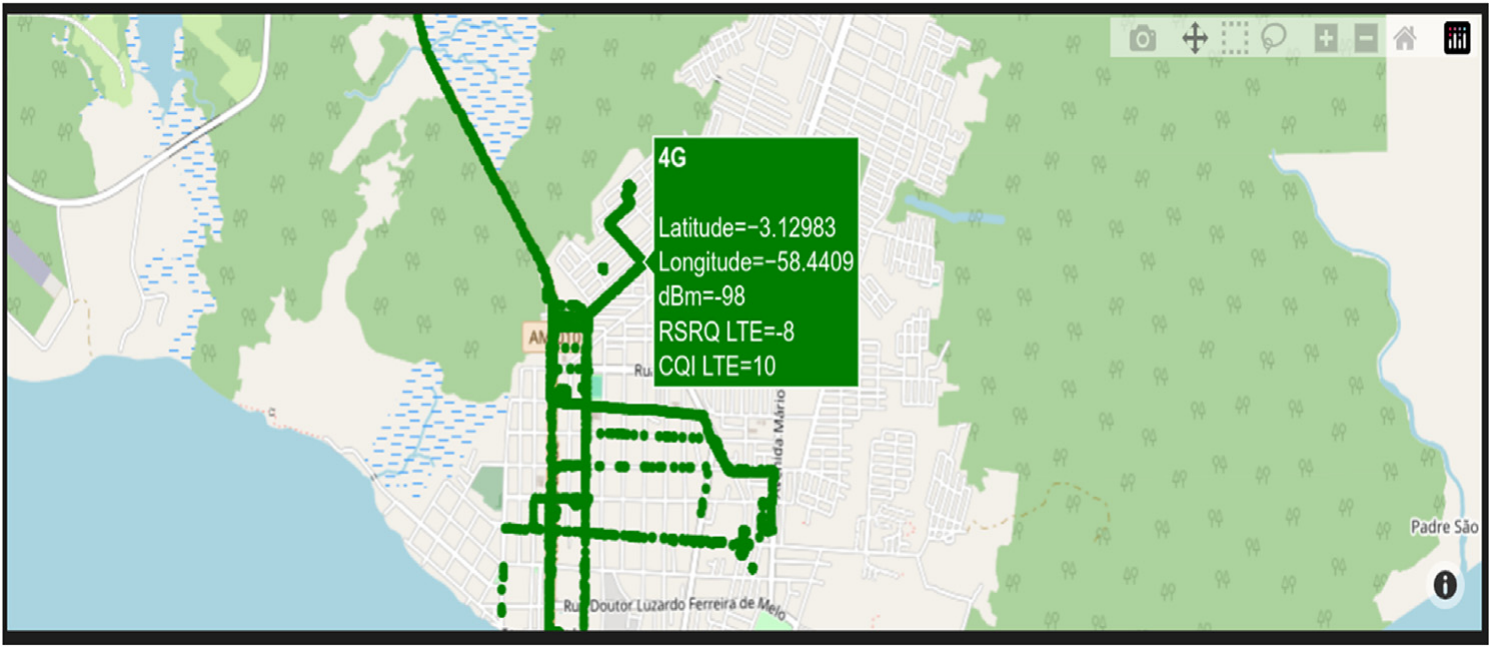
Plotting part of the geographic map covering the region of interest based on the RSRP LTE variable, this variable is essential to determine the quality of communication measured by the RSRQ LTE and CQI LTE variables.

**Fig. 5:**
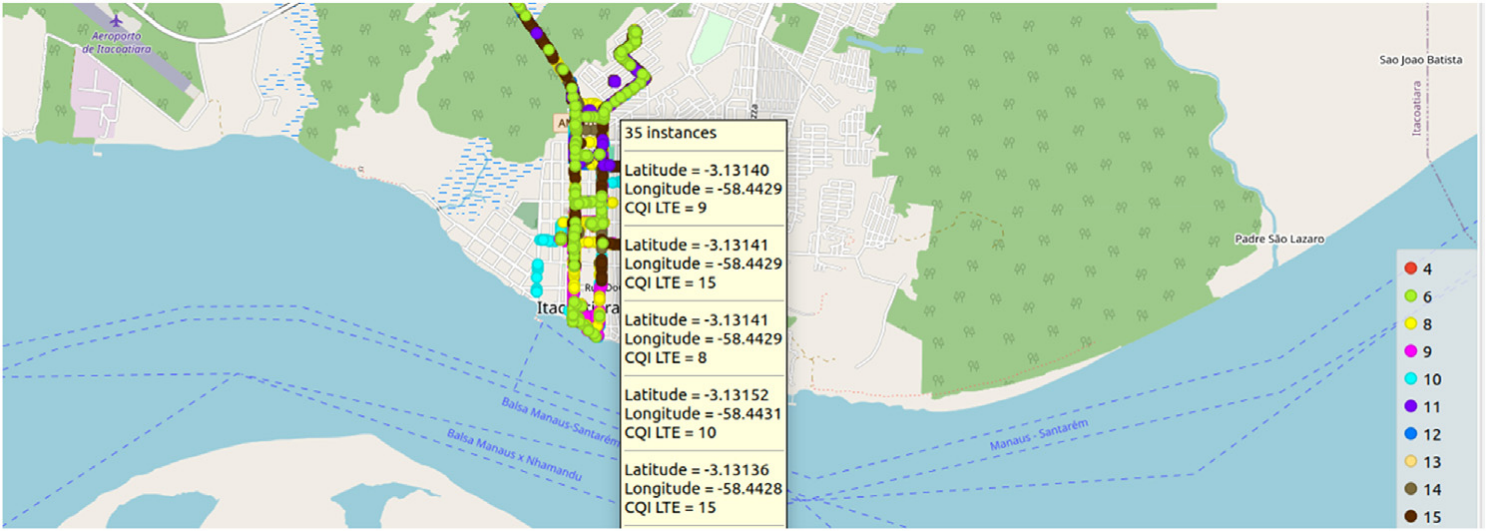
Plotting part of the geographic map covering the region of interest based on the CQI LTE variable; the larger the integer, the better the network quality; in this location, the prevalence was of the 4G LTE network.

From the pre-processed and anonymized data, it is possible to apply scripts to obtain insights into the dataset, apply analysis to the data, and generate various necessary graphs. Examples of graphs generated from the dataset are histograms, signal coverage location maps, and heat maps. Fig. 4, Fig. 5 illustrate an example of latitude and longitude-based graphs that can be generated from the data. In this case, the color of the graph represents the latitudes and longitudes traveled during the mobile device's path. The data presented in those figures corresponds to a portion of the path traversed by the mobile device in the city of Itacoatiara, Amazonas. It illustrates the technology employed in the collection, namely dBm, RSRQ, and CQI, which are utilized to assess the quality and coverage of the 4G LTE signal.

### 4.6 Collecting and exploring the dataset

**Fig. 6:**
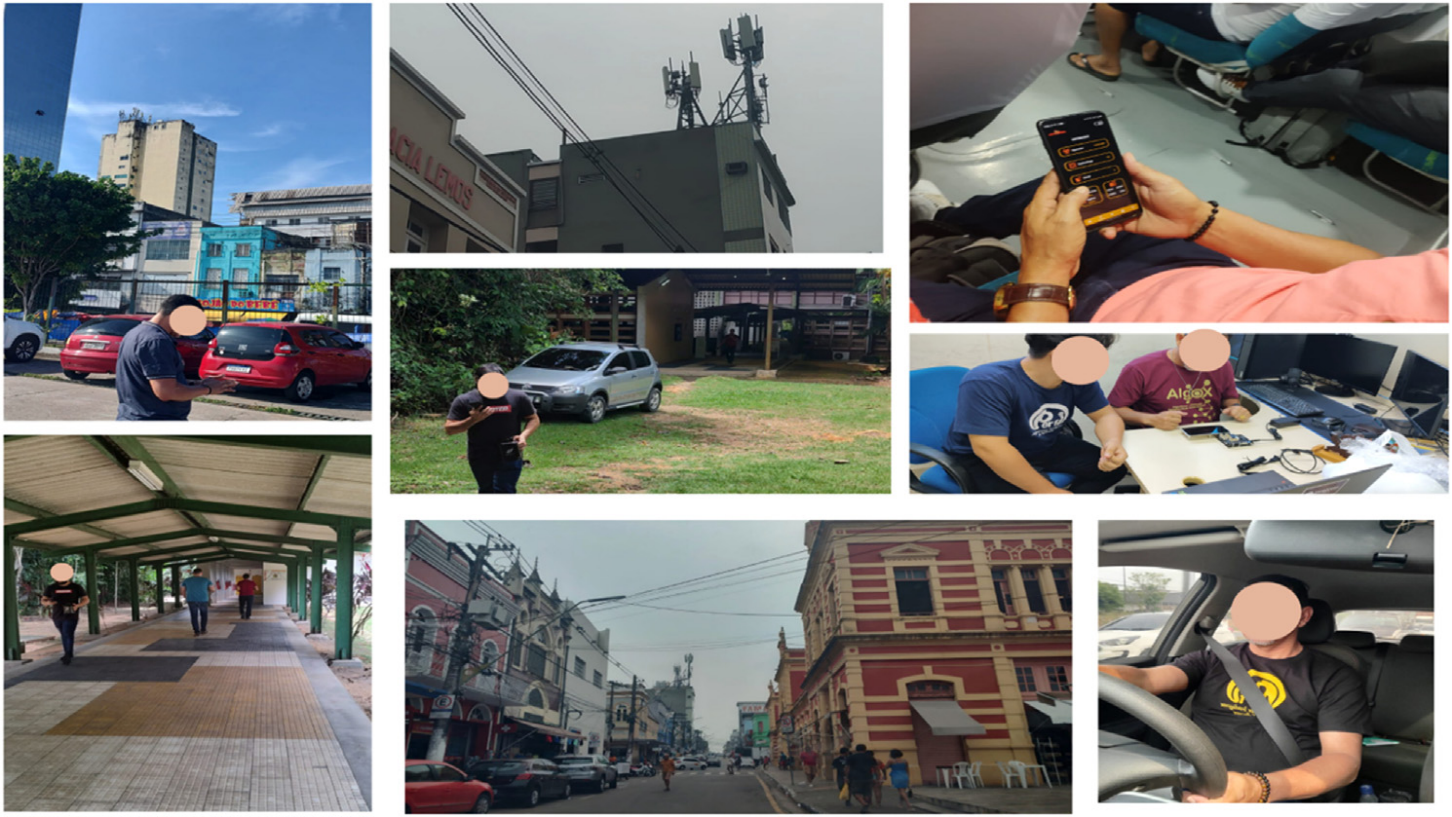
Volunteers collected data in different locations and modes. To verify that the collection was correct, the 4G LTE and 5G NSA antennas had to be visually mapped.

To validate the dataset, experiments were carried out using Python scripts, starting with data collection from mobile devices with Android OS from different manufacturers and the two main operators in the northern region of Brazil. Fig. 6 shows some specific collection points, comprising river and land transport modes, among which urban and rural areas stand out.

### 4.7 Performed experiments

**Fig. 7:**
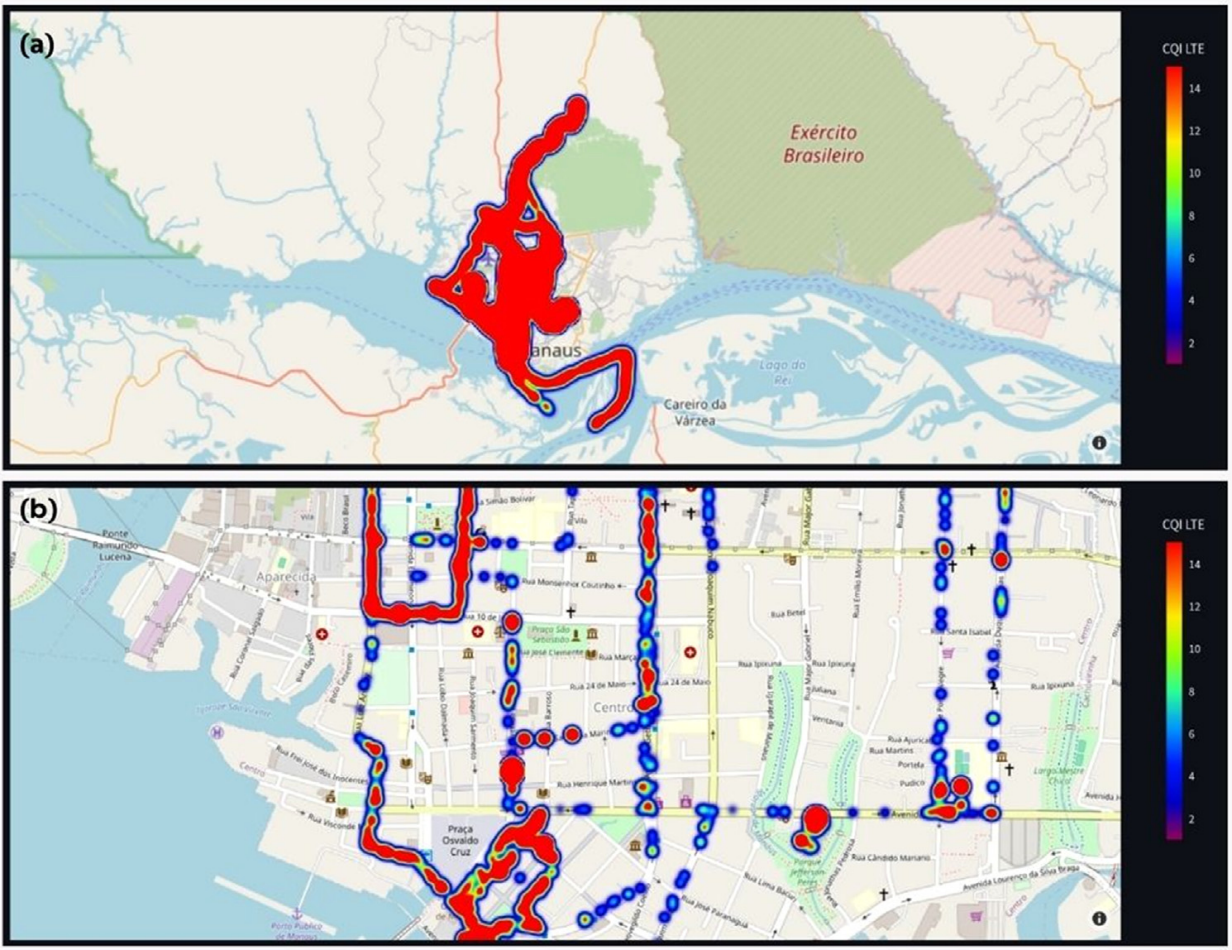
Heatmap plotting using the Pandas library, Plotly, and the Streamlit framework; (a) shows the total region of interest with its respective heat map; colors closer to red indicate the best CQI, while closer to blue indicate the worst. The scale on the right of the figure shows the colors and the respective CQI collected.; (b) shows the enlarged region of interest to evaluate the presented heat map better. In this case, (b) shows the central area of the city of Manaus, Amazonas.

Fig. 7(a) shows the experiments with heat maps. It presents the total region of interest based on the CQI LTE variable. In this map, the closer the color spectrum approaches red, “fire,” the better the mobile network signal quality. In Fig. 7 (b), we zoomed in on the map to better identify heatmap variations. The Python script used for this experiment is published with the dataset in the scripts folder. The heat map was created using data from the city of Manaus found in the “network_dataset” folder, in the file named “df_anonymizedManaus.csv”, with the heatmap.py script located in the src folder, both in the repository published on Mendeley. For this experiment, we used the Pandas libraries to generate data frames and graphs, as well as the open-source framework Streamlit [17] for developing data science and machine learning applications. We also used part of the database corresponding to the central region of the city of Manaus, Amazonas. To replicate the experiment, you need to have the Python interpreter along with the Pandas and the Streamlit library for designing the graphics.

In addition to the heatmap experiment, we generated some graphs to demonstrate the variety of uses of the dataset for the mobile network experiments we built. The scripts to generate the charts are published alongside the dataset in the scripts folder. They can be modified to create other charts of interest to other researchers in an open-source manner.

**Fig. 8:**
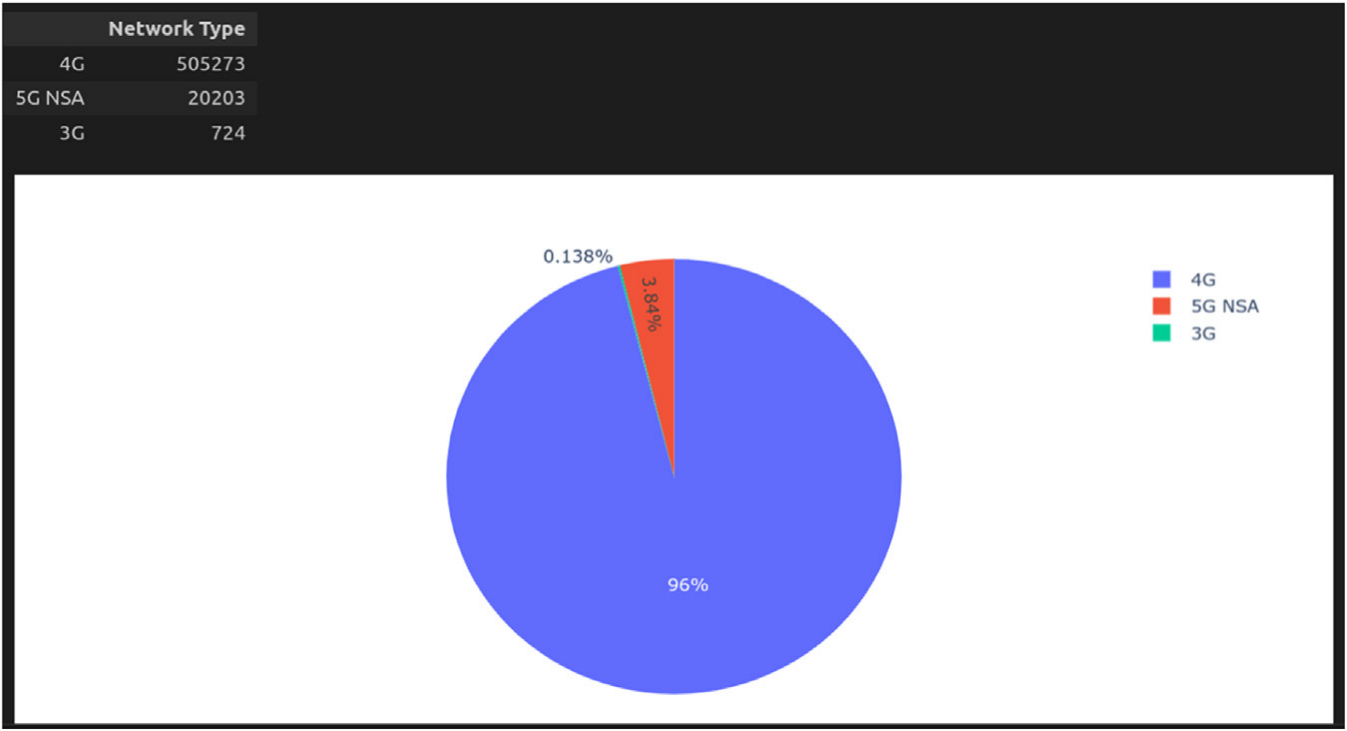
Pie chart evaluating 4G, 5G NSA, and 3G network technologies; at the time of collection, 4G still had the largest share, with 96% of occurrences.

Some insights we discovered with the dataset and exploration scripts were the signal quality concerning power, noise, and location and the 5G NSA coverage area in Brazil's implementation phase, which was still low at the time of collection. Fig. 8 presents a pie chart with data extracted from the base of the mapped region of interest. This graph shows that the amount of data collected from 4G technology is greater than that from 5G NSA technology. This indicates that the evaluated region predominantly uses 4G technology, and in some areas explored, 3G technology is still present. To replicate the experiment, you need to run the Python script graphics.ipynb, which is located in the src folder of the repository. You can use Jupyter Notebook or a similar application, such as Google Colab, for this purpose.

**Fig. 9:**
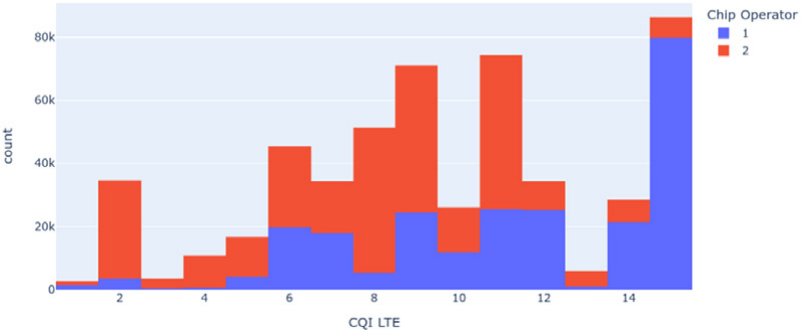
Histogram of CQI based at the Chip Operator.

**Fig. 10:**
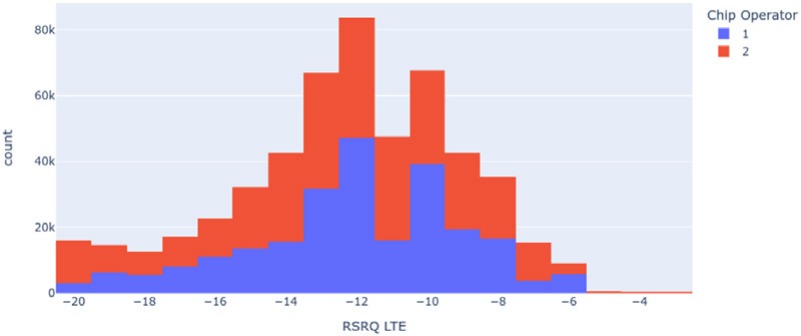
Histogram of RSRQ based at the Chip Operator.

Fig. 9, Fig. 10 present histograms generated to evaluate the quantity, balance, and quality of the data collected. In Fig. 9, it is observed that the operators, called operator 1 and operator 2 here to maintain the information's anonymity, have different CQI. In this case, we observe that the CQI levels of operator 2 are higher than those of operator 1.

Another critical insight obtained through the exploratory analysis of the dataset presented in this article is the operator and RSRQ relationship presented in Fig. 10. This variable is essential for evaluating signal quality and other correlated network variables. Here, we can see that operator 2 again has more RSRQ occurrences concerning operator 1, which corroborates the graph in Fig. 9, showing the relationship between the two network variables.

In addition to the conducted experiments presented in this article, it is important to note that the dataset can be used as input parameters for applying classic machine learning algorithms to assist in decision-making with the objective of categorizing the consumer experience. Since the dataset can be expanded, it can include other parameters of interest, such as the distance between the e-nodeB and the mobile device.

Classic Machine Learning algorithms can be employed to predict network quality characteristics. Among the available techniques are Linear Regression [18], Decision Trees [19,20], and Support Vector Machines (SVM) [21]. Using variables such as RSRP, RSRQ, RSNR, bandwidth (Band) and EARFCN (channel), Linear Regression can predict metrics like download speed or latency. This method estimates the relationship between the independent variables (RSRP, RSRQ, RSNR, Band, EARFCN) and the dependent variable, which could be a network quality characteristic such as CQI (Channel Quality Indicator). Similarly, decision trees can be used to categorize network quality into distinct levels, such as “Excellent,” “Good,” “Average,” or “Poor,” based on quality metrics and network characteristics. As a third example, the Support Vector Machine (SVM) algorithm can predict network service quality, such as the Packet Error Rate, based on the given variables.

Therefore, the dataset presented in this article is of fundamental importance for continued research in areas of low and poor mobile network coverage that compromises the quality of services by further study of the parameters that are critical for the quality of mobile data services, especially in regions with low/marginal signal coverage, which is the case of the Amazon region in Brazil.

Given that 4G LTE is the predominant technology in the metropolitan areas of the Amazon region, and its coverage is generally good only in densely populated zones, this can lead to performance issues in mobile units. Consequently, users may experience unstable and low-quality connections.

## Limitations

The dataset is confined to selected populous areas within Amazonas state, where 4G LTE and 5G NSA signals are available. However, Armadeira's data collection application is expanding its availability to North America and several Asian and European countries. It will substantially enlarge the dataset by incorporating new sites for exploratory analysis and algorithm application. Currently, the application supports only the Android Operating System, but a version for iOS is under development.

## Ethics Statement

The authors of this work declare that the paper meets the journal's requirements, guaranteeing that it is original and has not been submitted to any other vehicle. Furthermore, the research does not involve human subjects, animal experiments, or data collected from social media platforms.

## CrediT Author Statement

**Vandermi Silva, Carlos Freitas, Gabriel Tavares** Conceptualization, Methodology, **Vandermi Silva, Carlos Freitas, Gabriel Tavares and Felipe Maklouf**: Software Developer, **Vandermi Silva:** Data curation, Writing – Original draft preparation, Visualization, Investigation, **Rosiane de Freitas:** Project administration, Supervision, **Vandermi Silva, Rosiane de Freitas, Raimundo Barreto, Carlos Freitas, and Chavdar Ivanov:** Writing – Reviewing and Editing.

## Data Availability

Mendeley DataA multi-device and multi-operator dataset from mobile network coverage on Android devices (Original data).
